# Multimodal intervention to improve the transition of patients with inflammatory bowel disease from pediatric to adult care: protocol for a randomized controlled trial

**DOI:** 10.1186/s12876-022-02307-9

**Published:** 2022-05-18

**Authors:** Natasha Bollegala, Melanie Barwick, Nancy Fu, Anne M. Griffiths, Laurie Keefer, Sara Ahola Kohut, Karen I. Kroeker, Sally Lawrence, Kate Lee, David R. Mack, Thomas D. Walters, Jacqueline de Guzman, Claudia Tersigni, Ashleigh Miatello, Eric I. Benchimol

**Affiliations:** 1 Division of Gastroenterology, Department of Medicine, Women’s College Hospital, University of Toronto, Toronto, Canada; 2 Child Health Evaluative Sciences, SickKids Research Institute, The Hospital for Sick Children, Toronto, Canada; 3 Department of Psychiatry, University of Toronto, Dalla Lana School of Public Health, University of Toronto, Toronto, Canada; 4 Division of Gastroenterology, Department of Medicine, University of British Columbia, Vancouver, Canada; 5 SickKids Inflammatory Bowel Disease Centre, Division of Gastroenterology, Hepatology and Nutrition, The Hospital for Sick Children, 555 University Avenue, Toronto, ON M5G 1X8 Canada; 6 Department of Paediatrics and Institute of Health Policy, Management and Evaluation, University of Toronto, Toronto, Canada; 7 Division of Gastroenterology, Icahn School of Medicine at Mount Sinai, New York, NY USA; 8 Division of Gastroenterology, Department of Medicine, University of Alberta, Edmonton, Canada; 9 Division of Gastroenterology, Hepatology and Nutrition, BC Children’s Hospital, University of British Columbia, Vancouver, Canada; 10 Crohn’s and Colitis Canada, Toronto, Canada; 11 CHEO Inflammatory Bowel Disease Centre, Division of Gastroenterology, Hepatology and Nutrition, Children’s Hospital of Eastern Ontario (CHEO), Ottawa, Canada; 12 Department of Pediatrics, University of Ottawa, Ottawa, Canada; 13 ICES, Toronto, Canada

**Keywords:** Inflammatory bowel disease, Crohn’s disease, Ulcerative colitis, Pediatrics, Transition, Mental health, Implementation science, Randomized controlled trial, Health services research

## Abstract

**Background:**

Transition in care is defined as the “purposeful and planned movement of adolescents and young adults with a chronic medical condition from pediatric to adult-oriented healthcare systems/care providers.” Currently, there are no Level 1 evidence-based interventions to improve the care of transitioning adolescents and young adults (AYAs) with inflammatory bowel disease (IBD). The development of a transition program using a biopsychosocial approach will improve the standards for healthcare delivery to transitioning IBD patients. This is a protocol for a structured randomized controlled trial (RCT) to assess the clinical and implementation effectiveness of a multimodal intervention focused on improving patient function, transition readiness and outcomes among AYA patients with IBD being cared for at pediatric centers in Canada.

**Methods:**

This multi-center RCT is a type 1 hybrid effectiveness-implementation trial to evaluate effectiveness of the intervention and how it can be implemented more widely after the trial. We will include patients aged 16.0–17.5 years. The intervention program consists of 4 core components: (1) individualized assessment, (2) transition navigator, (3) virtual patient skills-building with a focus on building resilience, self-management and self-efficacy, and (4) a virtual structured education program. The control group will undergo standard-of-care defined by each participating center. The primary outcome will be the IBD Disability Index, a validated measure to assess patient functioning. Secondary outcomes include transition readiness and success, anxiety and depression scales, and health service utilization rates. Additionally, we will measure implementation outcomes and related barriers and facilitators for the intervention program.

**Discussion:**

The type 1 hybrid effectiveness-implementation design will allow for the development of a feasible, sustainable, and acceptable final intervention model. The intervention will consist of modules that can be accessed in an online, virtual platform. The implementation will allow centralization of interventions and funding in order to minimize the impact on local clinical practice or hospital resources. The authors anticipate that the main study limitation will relate to study subjects not completely adhering to every component of the intervention, which will be evaluated and addressed using the implementation science approach.

***Trial registration*:**

NCT05221281. Registry: ClinicalTrials.gov. Date of registration: February 2, 2022. https://clinicaltrials.gov/ct2/show/NCT05221281.

**Supplementary Information:**

The online version contains supplementary material available at 10.1186/s12876-022-02307-9.

## Background

### Transition from pediatric to adult care in adolescents and young adults with IBD

Transition in care is defined as the “purposeful and planned movement of adolescents and young adults with a chronic medical condition to adult-oriented healthcare systems/care providers” [1]. Children in Canada transition from pediatric to adult healthcare services between the ages of 14 and 18, with ultimate transfer to adult care by age 18. There are inherent differences between pediatric and adult care models; pediatric care is family focused, multidisciplinary, and has caregiver involvement for consent and guidance, while adult care is often a single provider and advocates for patient independence [2, 3].

Canada has amongst the highest rates of pediatric-onset inflammatory bowel disease (IBD) in the world [4]. The 2018 Impact of Inflammatory Bowel Disease in Canada Report [5] suggested that childhood-onset IBD is being diagnosed more frequently in Canadian children, increasing 50% in the first decade of the twenty-first century. Children with pediatric-onset IBD face unique challenges. Firstly, pediatric-onset IBD is more extensive, affecting more areas of the gastrointestinal tract [6]. The disease tends to be more inflammatory in nature resulting in lower surgical rates but greater need for immune suppressing medications to maintain remission [7]. As such, the direct costs of pediatric IBD to the health system are significantly higher [8]. In addition, living with a chronic disease can result in significant mental health difficulties in children and adolescents [9]. IBD, in particular, is associated with anxiety and depression [10, 11]. Finally, the onset of disease occurs at a particularly sensitive developmental period in the life of a child. The patient is tasked with developing a sense of self and independence from family while managing a difficult chronic disease [7]. This can interfere with typical developmental milestones that are important to support transition skills [12, 13].

In Canada, care to children and adolescents with IBD is almost exclusively provided by pediatric gastroenterologists, most of whom are affiliated with academic pediatric hospitals [14]. Many adult gastroenterologists have solo or group-based practice with limited multidisciplinary support [15]. Despite transition for adolescents and young adults (AYAs) being identified as a health services priority area [16], there is no standardization for transitioning adolescents with IBD in Canadian pediatric centers.

In light of the medical, psychosocial and health system burdens placed on AYAs with IBD during their transition from pediatric to adult care, an effective transition program could address the unique challenges described above and improve overall outcomes.

### The problem of implementation

While the methodology used to conduct clinical research has advanced, our ability to implement evidence-based practices has not kept pace. Approximately half of evidence-based practices ever reach widespread clinical use [17]. Implementation science aims to bridge the gap between the research-based demonstration of intervention effectiveness and effective and sustainable implementation in clinical practice or community settings. The proposed study is a *Hybrid Type 1* design that assesses both the effectiveness of the implementation strategy, and the health impact of the intervention [18]. *Hybrid Type 1* designs are typically used when the evidence for the effectiveness of the intervention is weak, and test the health impact of the intervention while explicitly collecting data on the implementation process to facilitate subsequent implementation efforts. The proposed study is a *Hybrid Type 1* design, acknowledging the lower quality evidence to support intervention in transition-aged patients with complex chronic disease while understanding the need for more robust data pertaining to IBD.

## Methods/design

This manuscript describes a protocol for a randomized controlled trial (RCT). We have reported details as per the SPIRIT reporting guidelines for RCT protocols [19].

### Purpose

**Aim 1** We will develop and evaluate the clinical effectiveness of a multimodal intervention consisting of 4 core components:

1. Individualized assessment of the AYA’s biopsychosocial needs.
2. The involvement of a transition navigator to facilitate the transition process and further identify and act upon the needs of the individual patient.
3. Patient skills-building exercises to improve areas of need, with particular focus on resilience, self-management, and self-efficacy.
4. Structured online eLearning program to build patient knowledge about the transition from pediatric to adult care and living with IBD as a chronic disease.

**Aim 2** We will evaluate implementation outcomes and contextual factors that hindered or facilitated delivery of the intervention to inform subsequent scale up of the intervention.

### Study design

The proposed study is a multi-center unblinded RCT of AYAs with IBD transitioning from pediatric to adult care. The intervention will consist of 4 core components while the control arm will be the typical approach to transition currently used in each participating Canadian IBD center. Participating sites include: The Hospital for Sick Children (SickKids)(Toronto, Ontario, Canada), Children’s Hospital of Eastern Ontario (CHEO)(Ottawa, Ontario, Canada), BC Children’s Hospital (Vancouver, British Columbia, Canada), and McMaster Children’s Hospital (Hamilton, Ontario, Canada). Additional sites may be added at a future date.

### Participants

**Fig. 1:**
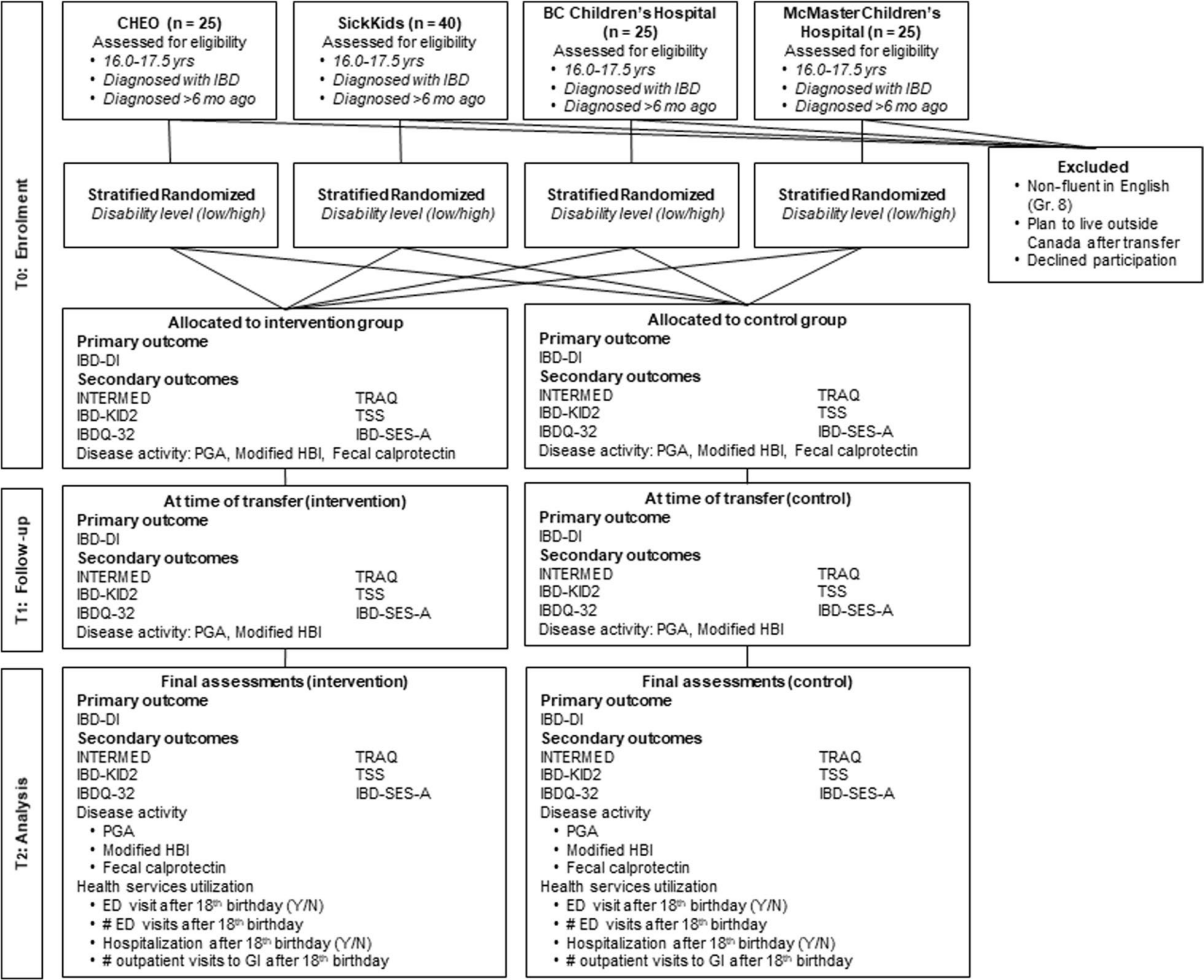
Patient flow

See Fig. 1 for patient flow.

Inclusion criteria: We will enroll adolescents aged 16–17.5 years with IBD diagnosed > 6 months before recruitment using standard criteria [20]. Since most adolescents are transferred to adult care around 18 years of age, this would allow for at least one year of post-transfer follow-up due to the 3-year length of the grant funding. Study participants must have the ability to speak/read English at a functional (Grade 8) level, intention to reside in Canada after transfer to adult care, and ability to use a smartphone or personal computer for the virtual intervention components. In Canada, > 95% of adolescent IBD patients in Canada have access to technology to complete study requirements [21, 22].

Exclusion criteria: Adolescents who do not fluently speak English, those intending to leave Canada after graduation from high school, and those unwilling to be involved will be excluded. We will maintain a record of ineligible patients, and those who declined enrollment and the reasons for doing so, to inform the feasibility of post-RCT implementation in clinical practice.

### Withdrawal criteria

It is not anticipated that any adverse events will occur as a result of study intervention. Participants who request study withdrawal will be removed and a withdrawal interview conducted to understand the reason for their study withdrawal. Non-adherence to the study intervention will be part of the data collected and analyzed and will inform program rollout.

### Setting

Participants will be recruited from pediatric IBD centers that are part of the Canadian Children IBD Network (CIDsCaNN). Participants will be followed past transfer of care and, thus, the intervention will follow AYAs during their care by adult gastroenterologists. Since the intervention is decentralized and virtual, no resources will be required from the receiving adult gastroenterologists. Adult providers (gastroenterologist and family physicians) will be given clinically relevant updates on the participants in their practice by the transition navigator (core component 2). At the end of follow-up, the adult provider will provide a summary measure of disease activity (see Outcome Measures).

### Randomization, concealment of allocation, and blinding

A subgroup of treating healthcare providers (physicians and nurses) will solicit interest in the study during their clinic appointments and based on clinic lists of their eligible patients compiled by research staff. The program is not being trialed with all IBD clinicians at all sites; this is appropriate for a Hybrid 1 design. Willing participants will then be consented by research staff who will also randomize them at enrollment (1:1) by research staff using two strata: center of care and IBD-DI severity (no/mild disability (scores of 0–34) vs. moderate/severe disability (scores of 35–100)) to ensure balance between the intervention and control arms in those strata. Randomization will occur directly in the REDCap (Vanderbilt University, Nashville, TN) database using the randomization function [23] and allocation will be concealed. Blinding is not possible as the transition navigators need to interact directly with the participants and their treating healthcare teams.

### Interventions

Intervention Group: Participants assigned to the intervention group will receive a multimodal intervention consisting of four core components.

#### Core component 1: individualized assessment

Each enrolled patient will undergo an individualized assessment of their biopsychosocial risk profile (Pediatric IBD INTERMED [24]), self-efficacy (IBD-SES-A) [25], functioning (IBD-DI) [26, 27], transition readiness (TRAQ) [28] and IBD knowledge (IBD-KID2) [29], administered by the transition navigator (see below) or research staff. In addition, supplemental questionnaires will be administered to assess depression, anxiety, and activation (i.e., a participant’s level of engagement in their own care); these will be self-administered by the participant with subsequent review by the transition navigator (Table 1).

**Table 1:** Tools used for individualized assessment and corresponding domains

Core skills	Questionnaire	Domains
*Main questionnaires*		
Coping/self-regulation*	IBD Disability Index (IBD-DI)	Functions, Activity participation, Structures, Environmental factors
Comprehensive	Pediatric IBD INTERMED	Biological, Psychological, Social, family/caregiver, Health systems
Assertiveness/self-efficacy	IBD Self-efficacy Scale for Adolescent (IBD-SES-A)	Self-confidence, Health-related quality of life
Tasks/knowledge	Transition Readiness Assessment Questionnaire (TRAQ)	Self-management, Self-advocacy
	Inflammatory Bowel Disease Knowledge Inventory Device Version 2 (IBD KID2)	Knowledge
*Supplementary questionnaires*		
Depression	Patient Health Questionnaire-9 (PHQ9)	
Anxiety	Generalized Anxiety Disorder-7 (GAD7)	

The INTERMED, PHQ9, and GAD 7 may be repeated as needed by the navigator to establish progress and update participants’ risk profile

The Pediatric IBD INTERMED is a validated instrument that assesses five domains of health (biological, psychological, social, family/caregiver and health system) reflecting historical/developmental, current state and future prognosis/vulnerability [24]. The instrument is is designed to facilitate inter-professional clinical communication and should identify issues related to the different domains of health. Identified psychosocial needs will be addressed by the care team, and/or in the skill-building component. The Pediatric IBD INTERMED and supplemental questionnaires may be repeated as determined by the navigator to establish progress and update the patient’s psycho-social risk profile.

All questionnaires will be administered at study enrollment, the time of transfer to adult care, and intervention completion.

#### Core component 2: transition facilitation with a navigator

Participants will be assigned one of two project-based transition navigators. The navigator roles will be staffed by a healthcare provider (nurse, nurse practitioner, or social worker) with knowledge of IBD, an understanding of the care pathway involved in transitioning IBD patients, and the skills and ability to provide psychosocial support.

The transition navigators will:

1. Administer the individualized assessments (core component 1) and identify AYA needs and gaps in knowledge.
2. Identify ‘red flags’ (e.g., suicidality, severe active symptoms/signs of IBD) requiring immediate physical or mental health intervention using the results of the Pediatric IBD INTERMED and the HEEADSSS psychosocial interviewer technique [30–32] and refer these issues to the patients’ healthcare team or other relevant professionals for potential management.
3. Engage in personalized skills-building exercises with the AYA, and/or refer them on to skills-building virtual activities (*core component 3*).
4. Establish goals of transition with the AYAs and their family/caregiver-guardians (*core component 3*).
5. Facilitate access and completion of the eLearning curriculum (*core component 4*).
6. Act as a central point of contact to answer the AYA’s questions, address concerns, ensure the AYA adheres to appointments and medications, and helps the AYA navigate the health system effectively.

The navigators will work remotely with the AYAs and their families, engaging via the AYA’s preferred technology: video teleconferencing, instant messenger (IM), telephone. The frequency of contact with the navigator will be driven by the participant, with a minimum number of contacts determined as twice yearly for most of the follow-up period, and two additional meetings in the year around transfer (6 months before and after the transfer date). At each visit, the navigator will complete a standardized Case Reporting Form (CRF) using REDCap to document discussions, assessments conducted, and a clear plan for intervention and/or discussion at the next exchange.

The interventions administered by the transition navigators and timeline are described in Table 2.

**Table 2:** Timeline and structure of the intervention provided by the transition navigator (*core component 2*)

Study time period	Activities of the transition navigator
Enrollment phase: T_o_ → 2 months after randomization	Pre-meeting with CD care team: This will consist of a case-review between the navigator and HCPs to review the AYA’s medical and psychosocial history, describe the plan for transition and establish a coordinated care pathway to be facilitated by the navigator. Among AYAs with an uncomplicated history, this review and planning could be done by email; otherwise, video teleconference software will be used Introductory meeting with the AYA and guardians/caregivers: The navigator will summarize the care pathway, the individualized goals of the intervention and answer basic questions about transfer to adult care. The navigator will establish SMART (Specific, Measurable, Achievable, Realistic, Time-based) goals for the family and AYA Second meeting with AYA alone: This meeting is intended to build trust, establish the limits of confidentiality, methods of communication, and boundaries. The navigator will propose SMART goals which may be distinct from the goals established with the family Meeting for individualized assessment: The aforementioned assessment tools will be administered (Core Component 1). Note: At the AYA’s discretion, this meeting may be combined with the meeting with the AYA alone and/or the meeting with guardians/caregivers
Maintenance phase: 2 months after randomization→ 1–2 years after transfer	The frequency of the virtual meetings during the maintenance phase will be flexible, with a minimum of 2 visits per year, depending on the needs and willingness of the AYA In the year around transfer (from 6 months prior to transfer, to 6 months after transfer), a minimum of 3–4 meetings will be planned A detailed script will be provided to the navigators to ensure a standardized list of topics are covered. The navigator will also work with the AYA and healthcare team to address deficits based on the AYA’s answers. Example questions to be asked by the navigator include: How are you finding the quality of your health care? How are things going with the biologic infusion clinics and the Patients Services Program (if applicable) How are you filling your prescriptions? How are the cost of your prescriptions covered? Do you know how to work with your insurance company to ensure your medications are covered? How did your visit with the doctor go? Did you parents go with you? How was your visit with the adult doctor? Did you find her/him approachable and knowledgeable? Did you feel you had enough time with the doctor? Did the adult doctor talk to you about colonoscopy, and how that might differ from colonoscopy in a pediatric hospital? What are you plans for post-secondary school work/university/college? Have you investigated your university’s disability program, and any accommodations to which you might be entitled because of your IBD?
Conclusion phase: end of study	Ongoing feedback of clinical and psychosocial concerns with adult GI Intervention completion will depend on patient readiness and the success of the skill-building exercises (*Core Component 3*), typically 1–2 years after transfer depending on age at enrollment Final assessments will be administered by the navigator at the final visit (*Core Component 2*), and the AYA will be instructed on how to complete the outcome measures

#### Core component 3: participant skills-building

The skills developed in the transition program will focus on the overarching goal of resilience, “one’s ability to bounce back from obstacles or adapt to change” [33]—in this case transition. This will be achieved by focusing on the following contributory skills: assertiveness, self-regulation, and improved confidence in individualized disease specific tasks (such as medication management, appointment keeping, tracking health issues and managing daily activities).

Mental healthcare providers in our group (LK, SAK) have already developed a standardized curriculum pertaining to these behavioral skills using cognitive behavior therapy (CBT) as an underlying framework (Table 3) [34]. These materials will be delivered virtually to all participants in the intervention arm. There will be mandatory assigned skills-building curriculum modules as well as optional modules to be assigned, as needed, by navigators. Transition navigators will be trained as motivational coaches and will lead separate personalized virtual sessions targeting individual skills that have been identified as lacking during the assessment phase. Successful skills-building curriculum completion will be defined as completion in ≥ 80% of the mandatory components.

**Table 3:** Skills-building methods to be used for specific skill deficits

Skill deficit	Skill-building methods
Deficits in emotional self-management domains (assertiveness and self-regulation)	CBT-informed, experiential pre-recorded video intervention created and recorded by psychologists (LK, SAK). In addition to core mandatory curriculum, additional experiential videos will be made to address deficits in: assertiveness, advocacy/communication, self-regulation and relaxation, and ability to manage pain, fatigue, overthinking, and social relationships.to ensure fidelity across sites
Deficits in assertiveness/self-confidence	10 modules that can be chosen based on need focused on assertiveness, symptom management (pain, fatigue, sleep) and mastering mental health (worry, mood)
Deficits in self-regulation	4 required modules focused on self-regulatory skills of disease acceptance, optimism, coping
Deficits in task-oriented self-management domains	Behavioral skills training provided by the transition navigator (e.g., self-injections, how to order a taxi to get to an appointment, how to refill a prescription)
Deficits in emotional self-management domains	Motivation and troubleshooting of barriers between visits provided by the transition navigator. Assignment to elective modules as appropriate

The transition skills-building program will prioritize high-needs individuals, as determined in *core component 1*. After baseline assessment, the skills-building curriculum will be tailored to the youth’s needs and developmental level as assessed by the Pediatric IBD INTERMED, TRAQ and IBD-SES-A. Skills-building materials will be delivered via online pre-recorded videos that first teach skills with an experiential component. There will be a basic training package of modules to be completed within 3 months of enrollment. The navigators will support any necessary needs and training and provide further individualization beyond the videos. The second year will focus upon completion of behavioral therapy sessions and progression to disease-specific task interventions with the navigator. Year three of the intervention will focus on the active reassessment of skills and targeted repetition of skill building activities as necessary. See Table 3 for interventions specific to various skills deficits.

#### Core component 4: structured educational eLearning curriculum

The aim of the education component is to provide consistent, accessible, and interactive information on key topics across 4 themes: healthy lifestyle, transitioning to adult care, self-care, and practical approaches to IBD-related tasks. The content is in development by the CIDsCaNN Education Committee and will be refined by experts, including adolescent medicine specialists, adult gastroenterologists, nurses, social workers, and other patients with IBD. eLearning modules will be created for each topic and AYAs living with IBD will advise regarding suitable formats for content delivery. A comprehensive manuscript detailing eLearning curriculum development is planned for future submission.

The navigator will provide participants with access to the curriculum, along with expectations for completion. Successful curriculum completion will be defined as completion of ≥ 80% of the mandatory modules. The eLearning modules will be piloted by the Patient Advisory Board (see below) and continuously improved based on feedback received from AYAs, the healthcare providers, and the navigators.

Control Group: The control group will receive the routine care transition supports currently available at each participating Canadian pediatric center. While every Canadian IBD center intervenes differently in transitioning AYAs, most have a semi-structured transition program. In order to standardize the control intervention, all participating centers will implement the following transition process:

1. A written letter explaining the goals of transition to the participants and their family.
2. Completion of age-appropriate checklists to ensure adolescents are meeting milestones of transition (developed by the TRACC Network) [35].
3. Online live educational webinars on transition and adolescent issues (once yearly, hosted by the CIDsCaNN Education Committee).
4. Individualized assessment (core component 1) at enrollment, the time of transfer, and study termination. Results of the Pediatric IBD INTERMED will be shared with the clinical team to provide intervention where indicated.
5. Completion of a transfer-of-care summary letter sent to the receiving adult gastroenterologist using a standardized letter template [36].

While the control group may also receive other transition supports currently in place in their participating care center, they will not receive the formal 4-component intervention described above. Their interaction with the navigators will be limited to completion of the INTERMED and other secondary outcomes measures.

### Evaluation of the implementation approach

The four core components of the intervention are defined as the essential functions that are hypothesized as necessary to produce outcomes in a typical service setting [37]. If the core components are not upheld, then the intervention will not function optimally.

Building on a large body of practice-change evidence in support of active rather than passive implementation efforts,^34,58^ we will guide each program site through a facilitated, staged, change process informed by implementation science. We expect that sites will vary somewhat in the timing of implementation activities. Sites will proceed through four stages of implementation based on the QIF/Meyers^59^ and described in detail below. Sites will create operational implementation teams that will execute implementation activities and report on implementation progress during monthly check-in-calls with the research team. Achievement of key implementation process milestones will be recorded, and monthly discussion calls will flag where more implementation support may be needed. The phases of our implementation process (Table 4) include:

**Table 4:** Summary of implementation process

Stage 1	Stage 2	Stage 3	Stage 4
0–3 months	0–3 months	4–24 months	24–36 months
Meet with leads at each site and navigators Review transition program practice profile	Create site implementation teams and meet monthly for coaching calls Identify and secure any necessary organizational supports/resources	Meet monthly with site implementation teams Gather process feedback, document implementation progress (strategies, challenges)	Meet with study sponsor to discuss scale up/national implementation Meet with site implementation teams to discuss full implementation at sites

Stage 1. *Initial Considerations for the Implementing Organization (to assess site capacity and need, build engagement):* The implementation researchers (MB, AM) will serve as implementation facilitators [38], meeting virtually with the leads at each implementing site to review the intervention practice profile that describes the 4 core components. Site leads will be invited to develop operational implementation teams of 3–4 people who will support site implementation activities. All will meet to discuss how core components will be delivered, by whom, and how we can ascertain that they were delivered with fidelity at each site. The practice profile enables effective implementation [37] as it ensures implementers understand the structures and functions required to deliver the intervention and have clarity on how they will facilitate delivery in their setting.

Site implementation teams will tailor a generic implementation plan using aspects of The Implementation Game© resource [39]. Adherence to the implementation process will be tracked and managed by site implementation teams with oversight by the implementation research team.

Stage 2. *Creating a Structure for Implementation* (occurs concurrently with initial recruitment of subjects and recruitment/training of transition navigators): Sites will explore all implementation drivers [40], the components of infrastructure needed to develop, improve and sustain the intervention as intended, as well as create an enabling context for the new way of working. Site implementation teams will work to put into place any necessary organizational supports (e.g., funding, human resources, new policies and procedures), including referral mechanisms, reporting frameworks and outcome expectations. Implementation teams will work together to manage organizational functions that can support implementation of the program. These activities will be informed by the Brief Checklist for Key Implementation Drivers [41]. As this phase begins, we will institute monthly implementation check-in calls to track progress, facilitate process and troubleshoot implementation barriers. Members of the implementation teams will be consented for surveys/interviews of their perceptions of the intervention and its implementation.

Stage 3. *Initial implementation and Evaluation of Implementation Quality*: Each site will begin delivery of the intervention. Process feedback and progress notes, and staff meetings will inform any refinements to the implementation and service delivery processes and address any staff support/skill building needs. To increase the fidelity of the implementation process across sites, we will use a checklist derived from the Quality Implementation Framework to track adherence to implementation stages and activities that have been tailored to our program and context. Each site will document progress, strategies, and challenges in relation to these tasks. Here, we will be mindful of site-specific factors and population-specific factors (e.g., sex, gender, ethnicity, rural vs. urban, LGBTQ status) that may influence implementation. In addition to implementation process tracking data, staff feedback will be used to refine the implementation process in an ongoing fashion.

Stage 4. *Full Implementation and Sustainability***:** At this stage, the transition intervention should be partially embedded at the participating centers and managed with existing resources. Once effectiveness of the intervention has been demonstrated, sites will assess the feasibility of full implementation (with their IBD clinicians) and sustainment using internal or external resources. Planning for sustainability will be embedded throughout all stages to build capacity ongoingly and ensure the intervention can be fully implemented and scaled up in their site and in other sites.

### Outcome measures

See Table 5 for summary of primary and secondary outcome measures.

**Table 5:** Primary and Secondary Outcome Measures. Note: Primary and secondary outcomes will be measured at enrollment (T1), at the time of transfer to adult care (T2), and at the end of the study period (1–2 years after transfer) (T3), unless otherwise specified

Domain	Measure	Timing of administration
*Primary outcome*		
Disability and function	IBD-DI [26, 27]	T1, T2, T3
*Secondary outcomes*		
Transition readiness	Transition Readiness Assessment Questionnaire (TRAQ) [28] Transition Success Scores (TSS)	T1, T2, T3
Bio-psychosocial risk	Pediatric IBD INTERMED [24]	T1, T2, T3
Disease-related knowledge	IBD-KID2[29]	T1, T2, T3
Quality of life	IBDQ-32[57]	T1, T2, T3
Self-efficacy	IBD Self-Efficacy Scale—Adolescent (IBD-SES-A) [58]	T1, T2, T3
Mental Health	Patient Health Questionnaire-9 (PHQ-9) Generalized Anxiety Disorder-7 (GAD-7)	T1, T2, T3
Disease activity	Physician global assessment (PGA) of disease activity Modified Harvey Bradshaw Index (HBI) Assessment for CD Activity [44] Pediatric Ulcerative Colitis Activity Index (PUCAI) for UC Activity [45] Fecal calprotectin *(at enrollment and study termination only)*	T1, T2, T3 T1, T3
Health services utilization	Emergency department visit after 18th birthday (yes/no) Number of emergency department visits after 18th birthday Hospitalization after 18th birthday (yes/no) Number of outpatient visits to GI after 18th birthday	T3

#### Aim 1 (effectiveness of the intervention)

Primary Clinical Outcome: Functioning: We will use the IBD Disability Index (IBD-DI) summary score [42, 43], an ordinal variable that measures participant functioning as the primary outcome. IBD-DI was selected as a validated measure of overall disability, functioning, and health. The primary outcome will be measured 3 years after enrollment.

Secondary Clinical Outcomes: The secondary outcomes measure transition readiness and program success, assessment of the five domains of health with emphasis on biopsychosocial risk, mental health, quality of life, and self-efficacy. In addition, by determining a marker of inflammation (i.e. fecal calprotectin) and disease activity (i.e. Harvey-Bradshaw Index [44] or Pediatric Ulcerative Colitis Activity Index [45]), we will assess whether the intervention improves overall remission rates. See Table 1 for a list of secondary outcomes.

Health Services Utilization and Cost: Study data will be deterministically linked to provincial health administrative data health at ICES (Toronto, Canada) and PopDataBC (Vancouver, Canada) using provincial health card numbers. Health service utilization will be tracked through the transfer of care by linking to physician billing, hospitalization and emergency department databases. To assess the effect of a transition intervention program on health services utilization after transfer to adult care with the potential that the intervention provides health system cost effectiveness while maintaining good health will be an important determination.

#### Aim 2: implementation science

Implementation outcomes: A recent systematic review of implementation outcome measures informed our selection process [49]. We identified four measures [46] (see Additional file 1: Table S1). The first three implementation outcomes will be measured in the early phase of implementation, while the remaining one will be measured following delivery of the intervention.

Barriers and facilitators: We will explore the factors associated with implementation effectiveness as described in the Consolidated Framework for Implementation Research [47]. Their identification will further inform the interpretation of clinical outcomes and implementation planning for future scale up.

### Statistical analysis

The primary outcome is the IBD-DI ordinal measure at the end of the treatment period (1–2 years after transfer). We will compare groups using the Wilcoxon-Mann–Whitney test, and statistical significance set at P < 0.05.

Secondary outcome measures will be analyzed at study completion and interim time points by comparing participants in the intervention and control arms using Wilcoxon-Mann–Whitney test. In addition, repeated measures analysis will determine changes over time for the primary and secondary outcome measures at three timepoints (baseline, time of transfer, end of study).

Assessment of health services outcomes will be conducted by linking clinical to health administrative data. Likelihood of emergency department use and hospitalization after transfer will be compared using multivariable logistic regression analysis. Number of ED and outpatient visits will be determined using either Poisson regression or negative binomial regression. All models will include confounders reported in Canadian health services studies of IBD patients: age at diagnosis, age at transfer, sex, rural/urban household, and socioeconomic status.

Assessment of implementation outcomes will be determined using mixed methods (quantitative and qualitative) assessment, as described above.

Sample size calculation: The IBD-DI ranges in score from 0–100. The categories of disability are 0–20 (no disability), 20–35 (mild disability), 35–50 (moderate disability), and 50–100 (severe disability). In the original validation study of the IBD-DI, the mean score of patients was 35.3 ± 20.5. We would consider the goal of this transition program to allow AYAs to achieve a mean level of < 20 (no disability), presenting a 16-point decrease from the mean baseline, or a Cohen effect size (*d*) of 0.78.

Assuming alpha-probability of 0.05, 46 patients would be required to achieve a power of 80% (23 in each group), and 62 patients would be required to achieve a power of 90% (31 in each group) (calculated using G*Power version 3.1.9.7). Assuming a drop-out rate of 25% in each group over the course of the study, we will aim to enroll 90 participants (45 in each group). This calculation is likely an underestimation of the study’s power. Two simulation studies have demonstrated that stratified randomization increases study power in trials with sample sizes < 100 [48, 49]. There is no established method for sample size calculation that accounts for stratified randomization [50]. We will limit enrollment to 40 participants from SickKids, and 25 from other sites in order to ensure distributed enrollment across sites and reduce the risk of health system bias.

### Ethical considerations and consent to participate

This study has been approved by the Research Ethics Boards of SickKids (1000078476) and CHEO (21/143X), and is under review by Research Ethics Boards from the other participating centers. In Canada, there is no ‘age of majority’ for medical treatment or research consent. Adolescents can consent to treatments and research if they are deemed competent and informed. Informed consent will be obtained from the adolescents by research staff. The navigators will discuss the limits of participant confidentiality with participants and their families, emphasizing that the five domains of health will be discussed with the healthcare team to initiate appropriate interventions.

### Data management

Study data will be managed by a study-specific data coordinating center. The data management team will be led by TDW and members of the data coordinating center of CIDsCaNN.

## Discussion

The incidence of childhood-onset IBD is rising, resulting in more patients undergoing transition from pediatric to adult care. Caring for patients during the transition process has been identified as a major issue in the health care of AYAs with IBD [51], and a failed transition jeopardizes the health of this fragile population. This study is the first RCT to evaluate a systematic and structured intervention to improve transition of IBD patients whilst studying its’ implementation.

A recent systematic review identified transition interventions reported in the literature in patients with IBD and other pediatric-onset chronic disease [52]. No RCT has been conducted in transitioning IBD patients. However, interventions reported in observational studies include transition clinics which include a multi-disciplinary team with adult and pediatric providers, educational programs which include self-efficacy and other skills-building, and structured transition programs [25, 53, 54]. RCTs conducted in transitioning adolescents with other chronic diseases are also sparse [52]. There are two active RCTs being conducted in Canada to evaluate transition navigators in adolescents with chronic diseases in Alberta [55], and group education in adolescents with type 1 diabetes [56]. These interventions were integrated as part of our plans for a multimodal intervention.

We acknowledge the complexity of running a multimodal intervention that aims to build transition related skills across a variety of healthcare situations (pediatric vs. adult care, multidisciplinary care pediatric care teams vs. single adult healthcare providers, regional and healthcare system differences, etc.) This presents unique operational challenges relating to the fidelity of intervention implementation. Centralization of the transition navigators, skills-building and educational modules is a targeted approach to offsetting these dilemmas. These navigators will be tasked with documenting and adapting their interactions to suit individual and local needs while preparing for the training of future navigators assuming the program were to expand.

While pediatric care has traditionally operated with a more fulsome allied healthcare team, adult IBD healthcare providers have mostly worked as single providers in a variety of settings from outpatient private offices to tertiary care IBD centers. Because many adult providers work in a community setting, their interaction and level of engagement with the interventions of this trial is hard to predict. We anticipate that transition navigators may have more challenges interacting with the adult team and for this reason the intervention arm should have a degree of independence and the necessity of well trained and experienced navigators to the current and future success of the program.

### Strengths

The primary strength of this study is that it is a collaborative but directed intervention which has the potential to meet the transition needs of a diverse group of patients being treated in a diverse variety of settings. The study builds off knowledge and experience garnered in other transition populations and the core components have been designed with the input of a multidisciplinary multi-specialty group of experts with high interest and experience in this area. There is an effort to study impact with regards to clinical outcomes, health resource utilization and economic cost. Finally, this study integrates an implementation science approach to ensure the fidelity with which the intervention is applied and the adaptive process which must occur at each local level to ensure its success.

### Limitations

Replicability may be perceived as a limitation of this RCT, considering the complexity of the intervention and specific resources required to implement it. In addition, the pragmatic nature of this RCT will allow for revision and improvement of the interventions to best suit the needs of the participating AYAs, which means the interventions are not static in time. Finally, our intervention may be specific to the context of the Canadian healthcare system. We will endeavor to publish details on the development and evaluation process of each component of the intervention in separate scientific manuscripts prior to the end of the trial period to allow for adaptation of the intervention to other health systems. However, we anticipate that the specific details of each component of the intervention are less important than the overall approach to intervention with a navigator-supported bio-psychosocial and educational framework. If the intervention is deemed effective in this trial, a quality improvement evaluative framework will be developed upon implementation of the intervention in clinical practice in Canada.

We anticipate that study participants’ program adherence is a possible limitation. As part of the Hybrid Type 1 design, we will monitor adherence carefully with detailed documentation provided by the navigator, and through activity/completion reports of the eLearning platform. We will determine why portions of the intervention were not completed and adapt the intervention at the time of widescale implementation to encourage fidelity. As part of this process, we anticipate that one or more components of the intervention may not be feasible. As we are measuring both clinical and implementation outcomes, we will be prepared to identify whether the failure is an *intervention failure* and/or an *implementation failure*. This knowledge will inform next steps prior to scale up in other settings.

Given that the intervention is strategically implemented over three years to ensure that age and developmentally appropriate skills are being reinforced during the transition years spanning pediatric into adult care, the concern regarding patient attrition is present. Given the typically high degree of engagement, parental oversight and multi-disciplinary involvement in pediatric care, the attrition risk is likely highest during early adult care. The transition navigators will be aware of this concern and use more provider-initiated interactions to sustain adherence and engagement.

The issue of sustainability is a concern. Healthcare administrators and patients have been involved to understand implementation challenges. The cost and cost-effectiveness will be monitored through health resource utilization. Finally, international groups must adapt and evaluate the intervention in the setting of other healthcare systems.

Finally, it is possible that the control group will have better results because of repeated research measurements, ongoing monitoring, and increased awareness of transition as an important health issue. We have attempted to design the control protocol to mimic standard practice but realize that monitoring bias may occur. However, this would reduce the risk of Type 1 error and provide more argument for wide implementation if efficacy is demonstrated.

## Conclusion

This multi-component prospective transition intervention study for pediatric onset IBD builds on the existing literature of observational research to improve patient outcomes, as well as RCTs in other pediatric chronic diseases. It offers a unique focus on implementation science and a credible opportunity for national and international dissemination of positive findings. When this RCT is complete, it will represent the highest-level evidence for a transition intervention for IBD patients.

## Supplementary Information

**Additional file 1**. **Supplemental Table 1.** Measures for Implementation Outcomes.

## Data Availability

Not applicable. Patient recruitment and data collection was not yet initiated at time of submission of this manuscript.

## Abbreviations

AYA: Adolescent and young adult;
CHEO: Children’s Hospital of Eastern Ontario (Ottawa)
CBT: Cognitive behavioral therapy
CIDsCaNN: Canadian Children IBD Network
CRF: Case reporting form
IBD: Inflammatory bowel disease
IBD-DI: IBD Disability Index
IBD-SES-A: IBD Self Efficacy Scale for Adolescents
IM: Instant messenger
PACE: Promoting Access and Care through Centres of Excellence
RCT: Randomized controlled trial
SickKids: The Hospital for Sick Children (Toronto)
TRAQ: Transition Readiness Assessment Questionnaire
