# Supplementary material for: Multimodal intervention to improve the transition of patients with inflammatory bowel disease from pediatric to adult care: protocol for a randomized controlled trial
Source: BMC Gastroenterol. 2022 May 18;22:251. doi: 10.1186/s12876-022-02307-9 (PMC9118697; doi:10.1186/s12876-022-02307-9)
Supplement: Supplementary file 1 — Additional file 1. Supplemental Table 1. Measures for Implementation Outcomes. [file 12876_2022_2307_MOESM1_ESM.docx]

## **SUPPLEMENTAL TABLE 1 (Online only). Measures for Implementation Outcomes.**

| **i)-iii) Acceptability, Adoption, and Feasibility**: will be assessed using the Hexagon Tool and the CFIR interview (described below). The Hexagon Discussion Tool[37] will be used with each sites implementation team to explore six contextual fit and feasibility factors, among them acceptability, intent to adopt, and feasibility. Together with the researchers, the site implementation team will review and discuss the questions for each indicator and document relevant considerations. Using the discussion notes and ratings, the team makes recommendations and confirms that they are willing to adopt the program or practice. While ratings are taken into account during the decision-making process, the ratings alone are not used to determine final recommendation to adopt the program. For analysis purposes, we will use the qualitative feedback to inform implementation planning. These meetings will be conducted virtually. |
| --- |
| 1. **Fidelity to the intervention and to implementation process:** For **Intervention Fidelity**, the unit of analysis will be the patient (n=x patients). The outcome will be percentage of completion for each of the four core components of the intervention: i) % of completed individual assessments; 2) % of completed scheduled Navigator meetings; 3) % of completed skills training; and 4) % completed core education modules. Completion metrics will be reported using percentages, means, medians, standard deviations, and interquartile ranges. A linear regression model will be used to identify predictors, with patient baseline characteristics such as sex, age, and illness complexity. The nesting of patients within each site will be accounted for in the analysis. All statistical tests will be two-tailed and statistical significance will be defined as p-values less than 0.05. **Implementation** **Quality** will be assessed using the aforementioned QIF-derived stage tracking form. |
| 1. **Penetration:** Penetration assesses the extent to which eligible program patients receive the intervention. This is important because a core aim of the program is to increase consistency of model delivery and access across all programs. Penetration will be calculated per site as a percentage; the number of patients receiving the program based on the number of patients who are qualified to receive the program. |
| 1. **Sustainability:** We will use the Sustainment Measurement System Scale [54] to assess 8 domains of sustainability: financial stability; responsiveness to community needs; responsiveness to community values; coalitions, partnerships, and networks; organizational capacity; organizational staff capability; implementation leadership; and evaluation and feedback and positive program outcomes. Developed for prevention programs funded by SAMHSA in the US, the SMSS can be used to monitor progress toward sustainment and provide feedback to stakeholders as to how to increase the likelihood of sustainment. Confirmatory factor analysis provided support for a 35-item model fit to the data. The SMSS (see appendix) will be adapted slightly to confirm to the language used in hospital clinical care. Analysis will include descriptive statistics for subscales and global score. A linear regression model will be used to identify predictors of global sustainability score, including site program fidelity and penetration. All statistical tests will be two-tailed and statistical significance will be defined as p-values less than 0.05. |
| 1. **Contextual Factors:** The Consolidated Framework for Implementation Research (CFIR) will be used to systematically assess contextual factors that are associated with effective implementation. The CFIR offers a comprehensive menu of constructs from published frameworks that influence implementation. The constructs are organized within five major domains: intervention characteristics (e.g., complexity, relative advantage); outer setting (e.g., external policy, patient needs); inner setting (e.g., resources, fit, leadership); staff characteristics (e.g., knowledge, beliefs); and implementation process (e.g., facilitation, planning, coaching). Similar to the Damschroder approach[55, 56] the CFIR will be used to develop a semi-structured interview to guide data collection. Interviews will be conducted with stakeholders at each site at the end of the active implementation (stage 3). The CFIR will provide the organizing framework for qualitative data coding and analysis, while being open to new, emergent themes. Participants from each implementing site will be consented by the research team and the interviews will be conducted by Dr. Barwick’s team at Sick Kids. Participant name and contact information will be sent to Dr Barwick’s team via SickKids secure file transfer network in order that interviews can be scheduled. ***Sample***. Interviews will be conducted with the two **navigators**, 3 members of the **implementation team** at each site, and 3 **patients** at each site for a total of 26 interviews. ***Procedures***. The interviews will be conducted by two analysts: an interviewer and a coder who captures notes in a templated interview form during the live interview. Following the interview, the coder will send the coded template to the interviewer who will review it to ensure accuracy. Both analysts provide an independent valence rating for each CFIR construct per interview (see below). ***Interview Protocol***. A CFIR interview protocol will be adapted to the study context based on CFIRguide.org. ***Analysis***. Using a modified rapid analysis (RA) approach means that data collection and coding occur together. The interviews will be conducted via 2-way video conferencing and will be audio recorded, but not transcribed, to provide an audit trail. The coder will capture the data directly onto a coding template that maps to the CFIR domains and factors as they are presented in the interview. The first column of the table identifies pre-specified “domains” based on the CFIR-informed interview guides. The second column is used to summarize key points from the interviews and to capture illustrative quotes. A third column captures valence (-2, -1, 0, +1, +2) for each factor. In the second stage of coding, factors and comments are synthesized by the unit of analysis (the site). The same template will be used to capture the summary of factors for a site, as well as the mode valence score for that site. Qualitative analysis will be supported with MAQXDA software. Audio recordings will be securely stored on Sick Kids secure servers, which are protected from unauthorized access by passwords. Audio recordings will be deleted after transcription and coding. Recordings, coded interviews (captured using the coding template) and MAXQDA file will be securely stored on Sick Kids secure servers for 7 years, as per TCPPS guidelines. Steps will be taken to ensure the confidentiality of participants’ information. All participants will be given a de-identified numerical code by the SickKids research team after consent. This de-identified code will be sent to SickKids in a password protected encrypted file via secure SickKids transfer network. Interviews will be audio recorded to check coding and rigor and deleted after coding. During audio recording of interviews, use of direct identifiers will be avoided. No personal information will appear in the coded CFIR templates. Quotes from interviews may be used in dissemination of the research findings, including conference presentations, and peer-reviewed journal articles, but no information will be included that might identify participants or program sites. |
